# Uterine Hemorrhage1Read at the thirty-second annual meeting of the Medical Association of Central New York, at Syracuse, October 17, 1899.

**Published:** 1900-04

**Authors:** A. L. Beahan

**Affiliations:** Canandaigua, N. Y.


### UTERINE HEMORRHAGE.[1]

By A. L. BEAHAN, M. D., Canandaigua, N. Y.

ABNORMAL hemorrhages from the uterus may result from distant or local causes, as pelvic congestion, uterine polypi, fibroids, malignancy, retention of products of conception, salpingitis, ovaritis and fungus degeneration of the uterine mucosa. Whether the hemorrhage results from these conditions, or is in close association with several conditions, is not easy to determine. It has been stated that the ovaries have nothing to do with the menstrual function; that the ovum is discharged independently of this period; that the hemorrhage is under the control of a pair of small nerves which enter the uterus under the Fallopian tube on each side and that cutting these would put an end to the monthly flow; that ganglia of nerves exist at the juncture of tube and uterus whose action is automatic and that unless the ligature is applied very close to the uterus when removing the appendages, uterine hemorrhage, either menstrual or irregular, will continue.

1. Read at the thirty-second annual meeting of the Medical Association of Central New York, at Syracuse, October 17, 1899.

The removal of uterine appendages for hemorrhages, due to uterine myomas of benign character, may be adduced as proof that either the ovary or tube, one or both, are directly related to this result. In uterine myomas the ovaries are often diseased and cystic, or fibroid degeneration has developed in the ovary, hence the relation of ovarian disease to uterine hemorrhage, is evident. Ovarian hyperemia induces menstrual loss and so becomes a frequent cause of anemia, the hyperemia becomes chronic, assuming an inflammatory phase, displacement of the ovary follows and finally impairment of health. The gross lesion in chronic ovaritis gives an increase of fibrous tissue at the expense of normal stroma, or cystic follicles form of the individual or multiple variety to such an extent that but little ovarian tissue is left. Either large or small cystic ovaries are provoking causes of severe and protracted hemorrhages, especially at the menstrual epoch. These hemorrhages may be equally severe with small cystic ovaries as with large. Regarding the relation of the Fallopian tube to uterine hemorrhages, we know that in ectopic pregnancies, if the rupture occurs at the outer portion or at the fimbriated extremity of the tube, surgical measures are demanded and the saving of life may be expected; but, if the tube is ruptured at the inner portion in proximity to the uterus, a fatal hemorrhage may at once occur. This fact is a proof of the theory already advanced as to the importance of the nerve or blood-vessels situated at or near the utero-tubal juncture. The influence of the general nervous system on uterine hemorrhage is very complex and the phenomenon we are now discussing is much influenced by its action.

The application of caustic, astringent, or stimulating remedial measures to the endometrium, if beneficial in checking uterine hemorrhage, must induce the result through the influence of the nerve reflexes, except in degenerative changes of the uterine mucosa, either benign or malignant. Even the curette, now so frequently used to lessen uterine hemorrhages, may exert its influence in the same irritative and reflex manner. Curettement is invaluable in its place when properly done, but the selection of cases requires careful judgment. The curette does not need to be of a stirrup hoe pattern or shaped like a ring fastened to the end of a rod, each made of steel, having sharp cutting edges. Nor does the operation require that the long strips of tissue be cut from the uterine cavity till it is reamed deeply into the normal tissue. Curettement is essential to remove retained placental débris, especially if organised, the granulations of chronic endometritis, for uterine polypi, or malignant degeneration. But in uteri that have never been disturbed by conception or where myomas exist and in cases having enlarged cystic ovaries, the curette is not indicated. It may do harm. A few cases may properly be related bearing on this subject.

Case I.—Patient, aged 30, single. In 1896 cervix was dilated and uterus curetted for dysmenorrhea and menorrhagia. These symptoms were better for a time, but in 1898 they returned. November 10, 1898, shortly after entering the hospital, had a chill with pain in left side, becoming general over the lower part of the abdomen. Pulse was increased and slight fever developed. November 11, twenty-four hours after the first attack, immediately following a rectal enema, shock developed, patient became pulseless, cyanotic and showed great nervous disturbance. Examination of the pelvis disclosed an enlarged left ovary and exquisite tenderness of the entire vaginal vault. Made abdominal section November 12th. Left ovary was large, cystic, containing old hemorrhages of tarry consistence. The right tube was in a state of acute inflammation, ovary cystic and loops of small intestine contiguous, were of pink color, as if exuded cyst fluid had caused a septic inflammation. Pulse and temperature became normal on third day; the incision wound healed by first intention and convalescence was uneventful.

The question arises, did the curettement in this case induce the hemorrhage in the cyst? If so, is there an intimate correlation between cystic ovaritis and uterine hemorrhages? The cysts by reflex action provoke uterine hemorrhages, while the curette in irritating the cavity of the uterus causes hemorrhages into the follicular cysts, giving small hemorrhagic infarctions, which may finally assume the appearance of cicatrices, or in the larger cysts causes dark, tarry deposits of partially inspissated hemorrhages.

Case II.—The patient, age 32, married, has had no children. In May, 1897, after protracted uterine hemorrhages, was curetted. The flow lessened for eighteen months, then increased, an enlarged appendix with general catarrh of large bowel, obstipation and gaseous distention were always associated in the condition. February 14, 1899, had sharp, serious uterine hemorrhage, became pulseless, face pallid, pupils dilated, surface of the body cold and clammy, in a condition of syncope. Diagnosis of diseased appendages and appendix was made. In a few days she was removed to the hospital, careful preparatory plans were adopted and March 13th, on abdominal section, both ovaries were found so full of cysts and hemorrhagic infarctions that their stroma was nearly destroyed. Appendix was enlarged, inflamed and contained a stercolith. Made clean removal. Pulse and temperature were always normal after operation and recovery was rapid. Health is now excellent.

Case III.—Patient, aged 30, has had three children; last one born August, 1898. Two miscarriages followed, the first in February, 1899, the second in June, 1899. Admitted to the hospital June 17, 1899. Gave history of fixed delusions since February last, with much excitement, mental symptoms exaggerated at menstrual epochs. Has been flowing much since last miscarriage and excessively for the last two years; is sleepless and worries constantly. It had been reasonably considered advisable to incarcerate in insane hospital. Pelvic examination shows subinvolutions of uterus, enlarged ovaries, especially the left; rectal ulcers and hemorrhoids with vaginismus. July 20th, operated for hemorrhoids and rectal ulcers with slight uterine curettage. Mental disturbance for one week less after which the delusions and excitement were intensified. Three weeks after the first operation, August 14th, an ovariotomy by abdominal section disclosed a dermoid cyst of the left ovary and cystic abscess of right ovary, the tubes on either side being remarkable for their length, which suggested possibility by their dragging of a reflex irritation with mental disturbance as a sequence. Recovery from operation was without a hitch and at the end of four weeks patient went to her old childhood home in the country. Delusions now are practically gone, excitement is abating, sleep is normal, appetite good, weight increasing and return of full mental vigor certainly is promised, though relapses may occur and the present outlook be delusive.

Case IV.—Patient, aged 32; married twelve years; miscarried soon after marriage. No other pregnancy until latter part of 1898. Menstrual hemorrhages always severe. Miscarried about January 10, 1899, after pregnancy of few weeks. In five days peritonitis with great distension developed. Examination showed enlarged ovaries, both tender. Abdominal section, January 24th. Ovaries cystic. Left ovary showed cicatrix of recent rupture of small cyst, with peritonitis, congestion of loops of intestine. Proof of cause of peritoneal attack. Right ovary full of cysts. One larger than the rest. Appendages removed. Recovery rapid.

These major reflexes with their hemorrhages are from without the uterus and uterine hemorrhage is thereby greatly influenced. In uterine myomas the degenerative changes in or upon the mucous membrane are insignificant and will not account for the associated bleedings, but the coexisting ovarian disease may be looked upon as the more probable cause. Local tinkering for uterine hemorrhages with suppositories, swabs of caustics, alteratives or astringents in which category may also be placed, the usual, if not the unusual, surely the uncertain, electrical currents of any and all forms, are unreliable and pernicious. Hot vaginal douches with alkaline or astringent solutions and saline cathartics may be depended upon to meet about all the medical demands and their action is certain. But other forms of medication as ergot, astringents, tonics and tissue builders will always find constant if unsatisfactory employment.

